# Genetic Variation Among Tropical Maize Inbred Lines from NARS and CGIAR Breeding Programs

**DOI:** 10.1007/s11105-022-01358-2

**Published:** 2022-09-26

**Authors:** Stella Bigirwa Ayesiga, Patrick Rubaihayo, Bonny Michael Oloka, Isaac Onziga Dramadri, Richard Edema, Julius Pyton Sserumaga

**Affiliations:** 1 Department of Agricultural Production, College of Agriculture and Environmental Sciences, Makerere University, P. O. Box 7062, Kampala, Uganda; 2 Makerere University Regional Center for Crop Improvement (MaRCCI), College of Agriculture and Environmental Sciences, Makerere University, P. O. Box 7062, Kampala, Uganda; 3 National Crops Resources Research Institute, National Agricultural Research Organization, P.O. Box 7084, Kampala, Uganda; 4 National Livestock Resources Research Institute, National Agricultural Research Organization, P.O. Box 5704, Kampala, Uganda

**Keywords:** Genetic diversity, Maize inbred lines, Population structure, SNP markers

## Abstract

The use of molecular markers allows for precise estimates of genetic diversity, which is an important parameter that enables breeders to select parental lines and designing breeding systems. We assessed the level of genetic diversity and population structure in a panel of 151 tropical maize inbred lines using 10,940 SNP (single nucleotide polymorphism) markers generated through the DArTseq genotyping platform. The average gene diversity was 0.39 with expected heterozygosity ranging from 0.00 to 0.84, and a mean of 0.02. Analysis of molecular variance showed that 97% of allelic diversity was attributed to individual inbred lines within the populations while only 3% was distributed among the populations. Both neighbor-joining clustering and STRUCTURE analysis classified the inbred lines into four major groups. The crosses that involve inbred lines from most divergent subgroups are expected to generate maximum heterosis and produce wide variation. The results will be beneficial for breeders to better understand and exploit the genetic diversity available in the set of maize inbred lines we studied.

**Supplementary Information:**

The online version contains supplementary material available at 10.1007/s11105-022-01358-2.

## Introduction

Maize (*Zea mays* L.) is a widely produced and consumed cereal crop, with worldwide production increasing from 313 million metric tons in 1971 to 1162 million metric tons in 2020 (Statista 2022). Maize was first domesticated in central Mexico approximately 9000 years ago (Xiao et al. 2016). It has since spread to every continent except Antarctica, making it one of the most abundant cereal crops worldwide for food, fuel, and feed (FAOSTAT 2013).

In sub-Saharan Africa (SSA), maize is the most important cereal crop as food, feed, and industrial crop, grown on over 40 million hectares of land (Cairns et al. 2021). The biggest percentage (approximately 67%) of the maize produced in developing countries comes from small-scale farmers, accounting for 30–50% of the low-income household expenditure in Eastern and Southern Africa (Chemiat and Makone 2015).

Determining the genetic diversity and relatedness among maize germplasm is an important step in maize improvement (Adekemi et al. 2020). Information on genetic diversity will assist in choosing the best breeding approaches, parental line selection, and expansion of the genetic base of maize germplasm in a breeding program (Ajala et al. 2019). This information is also vital for assessing how much the genetic diversity is lost due to conservation or selection (Badu-Apraku et al. 2021). Understanding the genetic diversity among maize inbred lines is important, as selecting and crossing genetically diverged parents can produce hybrids with high heterotic effects.

Assessing genetic diversity using phenotyping methods is costly and cumbersome, and the data could be unreliable because they are greatly influenced by the environment (Ajala et al. 2019). Molecular markers are, therefore, preferred for evaluating genetic diversity because they are polymorphic and stable and are not affected by environmental factors, and can handle a large number of lines. Various types of molecular markers have been used to evaluate genetic diversity and classify the maize inbred lines into their respective heterotic groups, including random amplified polymorphism DNA (RAPDs), amplified fragment length polymorphism (AFLPs), restricted fragment length polymorphism (RFLPs), simple sequence repeats (SSRs), and single nucleotide polymorphisms (SNPs) (Dao et al. 2014; Semagn et al. 2012; Sserumaga et al. 2019; 2020; Adekemi et al. 2020).

SNP markers are becoming popular for diversity studies because they are widely distributed and abundant throughout various crops’ genomes (Badu-Apraku et al. 2021). Several SNPs are now accessible in maize, and many of them have been developed from the DNA sequence of known genes; this makes them the best choice for various activities in maize improvement such as genetic diversity analysis (Dao et al. 2014). At present, DArTseq is one of the cheap and easy but efficient genotyping-by-sequencing platforms which allows genome-wide marker discovery through a restriction enzyme-mediated genome complexity reduction and sequencing of the restriction fragment, and also results in higher marker densities (Edet et al. 2018). Various studies have been conducted to determine the genetic diversity in maize germplasm using SNP markers, for example, Dao et al. (2014) studied the genetic diversity and patterns of relationships of 59 local maize lines developed at the Institute of Environment and Agricultural Research (INERA) in Burkina Faso and 41 exotic inbred lines using 1057 SNP markers. Wu et al. (2016) assessed a panel of 538 CIMMYT maize inbred lines (CMLs) and 6 temperate inbred lines using 362,008 SNPs. Sserumaga et al. (2020) studied the genetic diversity of 50 maize inbred lines with resistance to common rust.

Maize has very high genetic diversity because of its comprehensive selection, and it is a model crop for key cereals since its genome is known for tremendous phenotypic and molecular diversity (Adekemi et al. 2020). The aim of this study was to assess the genetic structure and genetic diversity of the tropical maize inbred lines using high-density single nucleotide polymorphism (SNP) DArTseq markers. This information will be useful to breeders in both the national breeding program (NARO) and CIMMYT for selecting parents for crosses and in determining appropriate conservation strategies.

## Materials and Methods

### Plant Materials, DNA Extraction, and Genotyping

A diverse panel of 151 maize inbred lines (Table S1) was used in the study, which included inbred lines developed from the National Agricultural Research Organization (NARO) in Uganda, and CIMMYT maize breeding programs.

A single seed of each of the 151 inbred lines was grown in a screen house at the National Crops Resources Research Institute, Namulonge, Uganda. At the 3–4-leaf stage, leaf samples were harvested following the leaf sampling protocol from LGC Genomics (http://www.lgcgroup.com/our-science/genomics-solutions/#.WXpE7ITyu70) using the plant sample collection kit from LGC Genomics (Sserumaga et al. 2019). These were then shipped for DNA extraction and genotyping at Integrated Genotyping Sequence Support (IGSS) platform found at Bioscience for East and Central Africa (BecA)-Hub, Nairobi, Kenya. Genotyping was done using DArTseq genotyping platform and a total of 35,054 SNPs were identified in the population. Quality control was done by filtering data using a minor allele frequency of 5% and a minimum count of 80% of the sample size, done in TASSEL v.5.2 software. Monomorphic SNPs and those that had heterozygosity of > 0.05 and missing data of more than 10% were discarded. After filtering and quality control, 10,940 SNP markers were retained and used for genetic diversity analysis.

### Diversity Analysis

For each marker, polymorphic information content (PIC), gene diversity, and allele frequency were calculated using PowerMarker version 3.25 (Liu and Muse 2005). The PIC value is the relative value of each marker with regard to the amount of polymorphism displayed (Lu et al. 2009). Gene diversity is the probability that two alleles randomly selected from the test sample are different; heterozygosity which is the fraction of heterozygous loci detected in each inbred line was also obtained (Lu et al. 2009). The genetic distance between the lines was calculated using Roger’s genetic distance (Rogers 1972). A dendrogram was generated from the genetic distance matrix with the neighbor-joining method in PowerMarker version 3.25 (Liu and Muse 2005). The resulting trees were visualized using MEGA version 11 (Tamura et al. 2011). Principal coordinate analysis (PCoA) based on the genetic distance matrix was obtained using GenAlEx 6.5 software (Peakall and Smouse 2006, 2012).

To assess the population structure of the 151 inbred lines, STRUCTURE software (Pritchard et al. 2000) was used with the 10,940 SNPs. The number of subpopulations was computed by setting the number of clusters (K) ranging from k = 1 to 10. Ten replicates for each *k* value were run using correlated allele frequencies and the admixture model (Falush et al. 2003). To determine the correct number of subpopulations using posterior probabilities (qK), a 100,000 burn-in period was used, as well as 100,000 iterations (Sserumaga et al. 2019). To estimate the most likely number of clusters within the population and the best K for grouping the inbred lines, the Evanno transformation method (Evanno et al. 2005) was used on the outputs obtained from STRUCTURE, where delta K was calculated for each value of K using the Structure Harvester software (Evanno et al. 2005). Each inbred line was assigned to a cluster when the proportion of its genome in the cluster (qK) was higher than a threshold value of 50%. In addition, principal component analysis (PCA) was performed with GAPIT in the R software (R Core Team 2021), to illustrate the genetic relationships among the 151 maize lines tested and compare different subsets of germplasm. Analysis of molecular variance (AMOVA) was run to compute the variance among the populations and accessions within populations using GenAlEx 6.5 software (Peakall and Smouse 2006, 2012).

## Results

### SNP Characterization, Genetic Distance, and Relationships

**Fig. 1:**
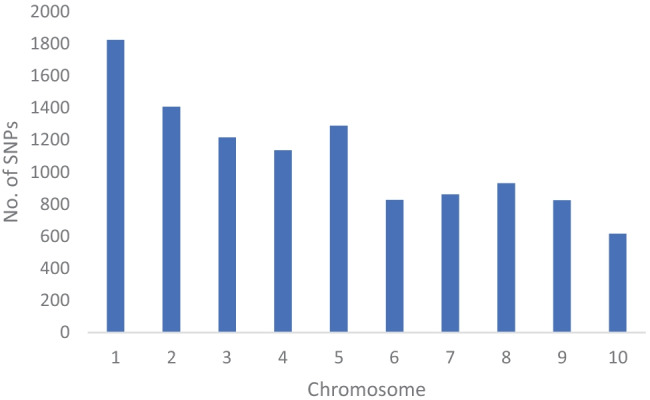
Number of SNP markers distributed across the 10 chromosomes

**Fig. 2:**
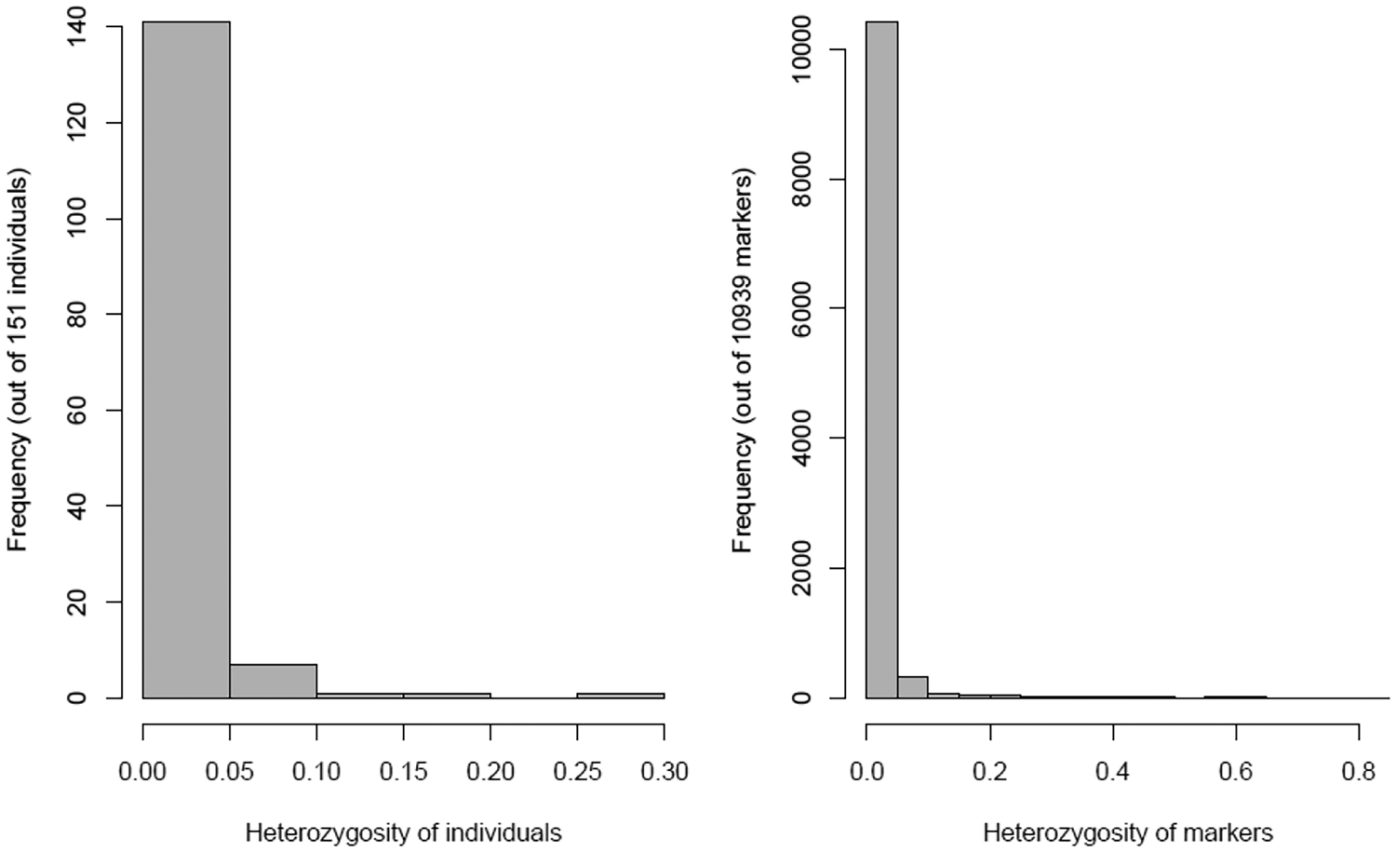
Heterozygosity rate among the 151 inbred lines and the 10,940 SNP markers

Distribution of the 10,940 SNP loci varied across the 10 chromosomes (Fig. 1), chromosome 1 had the largest number with 1826, and chromosome 10 had the least with 617 SNPs. The minor allele frequency ranged from 0.1 to 0.50 with an average of 0.26 and the gene diversity had an average of 0.39 ranging from 0.19 to 0.88, while heterozygosity, ranging from 0.00 to 0.84, had an average of 0.02. It is important to note that the biggest percentage of the inbred lines (95%) had a heterozygosity rate of less than 5% (Fig. 2). The PIC value had an average of 0.33 with the values ranging from 0.17 to 0.87, and the genetic distance ranged from 0 to 0.39, with an average of 0.31. The summary statistics attained from the SNP markers are shown in Table 1.

**Table 1:** Diversity indices statistics of the 151 maize inbred lines based on 10,940 SNP markers

	**MAF**	**GD**	**PIC**	**He**	**GDist**
**Maximum**	0.50	0.88	0.87	0.84	0.39
**Minimum**	0.10	0.19	0.17	0.00	0.00
**Average**	0.26	0.39	0.33	0.02	0.31

*MAF* minor allele frequency, *GD* gene diversity, *PIC* polymorphic information content, *He* heterozygosity, *GDist* genetic distance

### Cluster Analysis

**Fig. 3:**
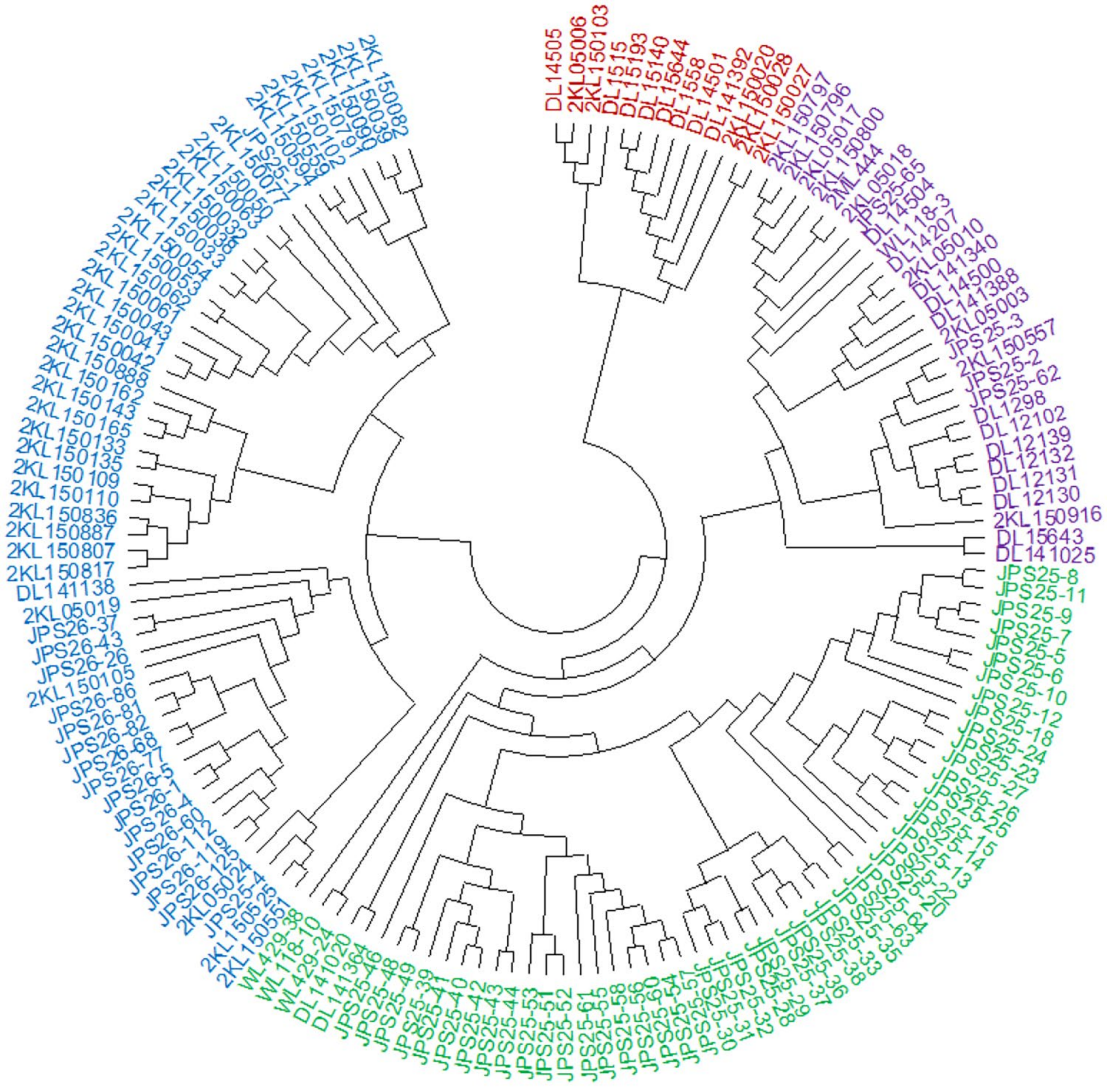
Neighbor-joining tree for 151 maize inbred lines based on Roger’s genetic distance

The neighbor-joining tree using the identity by state matrix was generated to illustrate the genetic diversity among the 151 inbred lines (Fig. 3). The dendrogram classified the lines into four major groups. Group 1 consisted of 13 (8.6%) inbred lines with two subgroups, group 2 had 28 (18.5%) lines in four subgroups, group 3 comprised of 55 (36.4%) lines in six subgroups, and group 4 also had 55 (36.4%) inbred lines in five subgroups.

### Population Structure

**Fig. 4:**
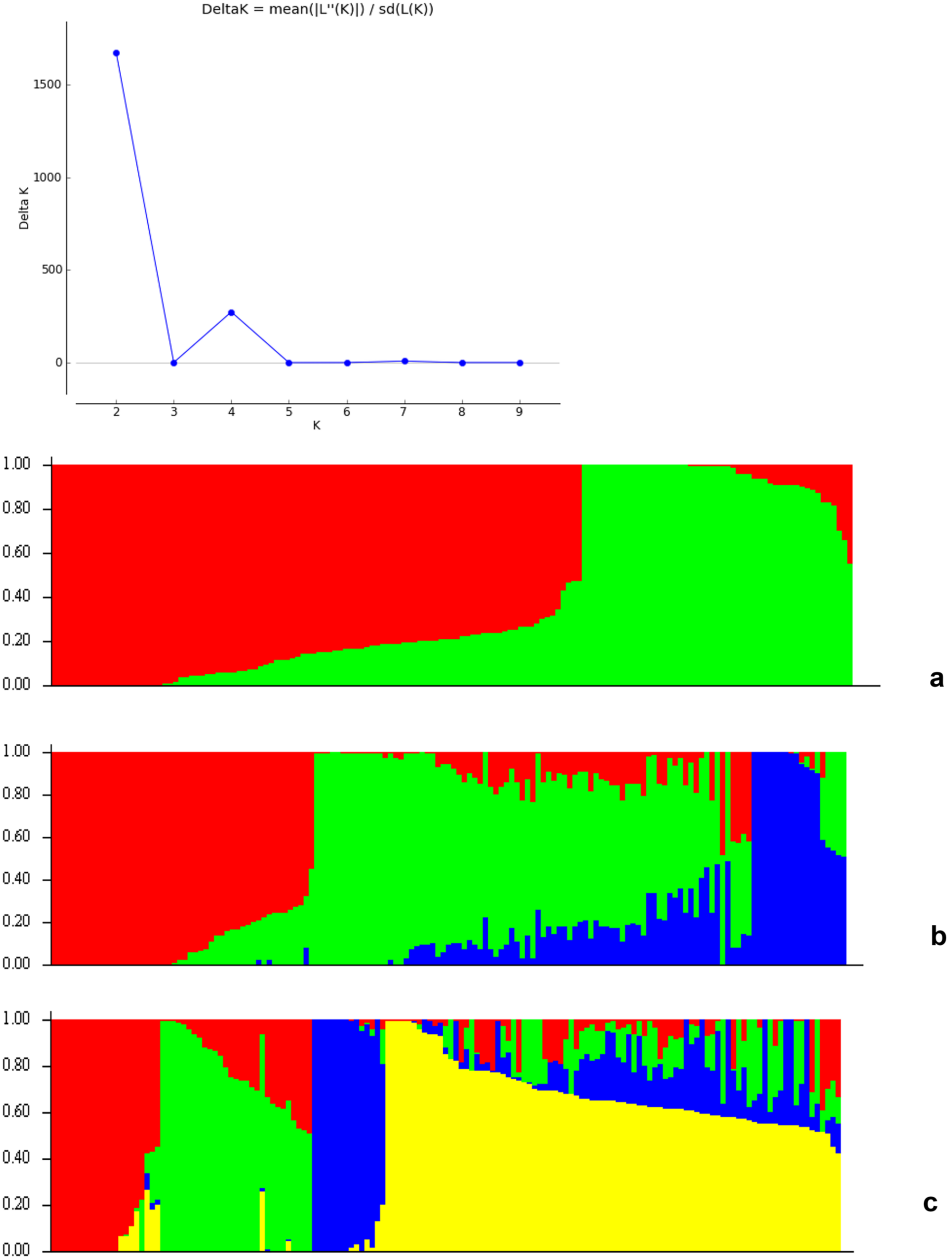
**a** Population structure among individuals with ΔK = 2. **b** Population structure among individuals with ΔK = 3. **c** Population structure among individuals with ΔK = 4. Each subpopulation is represented by a different color

Based on the (LnP(D)) and ΔK, the output from population structure analysis of the 151 inbred lines suggested the presence of either 2 or 4 clusters (Fig. 4). At K = 4, subpopulation 1 comprised of 14% (21 lines), 19% (29 lines), 11% (16 lines), and 56% (85 lines) in subpopulations 2, 3, and 4, respectively. Subpopulations 1 and 2 comprised of inbred lines obtained from NARO only, subpopulation 3 consisted of lines from CIMMYT, and finally, subpopulation 4, the most diverse group, was composed of all the different source populations. Although the outputs from STRUCTURE (Fig. 4) and those from NJ cluster analysis (Fig. 3) revealed four subpopulations, the inbred lines assigned in each of the four groups were quite different.

The expected heterozygosity among inbred lines within the four subpopulations ranged between 0.16 for cluster 1 and 0.30 for cluster 4 with an average of 0.22. The F_ST_ values for subpopulations 1, 2, 3, and 4 were 0.65, 0.50, 0.63, and 0.24, respectively. The allele frequency divergence values were 0.27 between subpopulations 3 and 1, followed by 0.23 recorded between subpopulation 3 and 2, then 0.17 observed between subpopulations 4 and 1, and the least allele frequency divergence of 0.14 recorded was between subpopulations 2 and 4, and 3 and 4.

### Analysis of Molecular Variance

The goal of AMOVA was to use SNP markers to assess population differentiation in the maize inbred line populations. The results of AMOVA revealed that differences between and within populations accounted for 3% and 97% of the variation, respectively (Table 2).

**Table 2:** Analysis of molecular variance for genetic differentiation among and with clusters of maize inbred lines

Source	Df	SS	MS	Est. Var	% Var
Among populations	3	12,412.67	4137.56	66.78	3
Within populations	147	296,035.76	2013.85	2013.85	97
Total	150	308,448.42		2080.63	100

Genetic differentiation among accession populations (PhiPT) = 0.0321; *P* = 0.0001. *P*-value is based on 9999 permutations

*df* degree of freedom, *SS* sum of squares, *MS* mean squares, *Est. Var* estimated variance, *% Var* percentage variation

### Principal Component Analysis

**Fig. 5:**
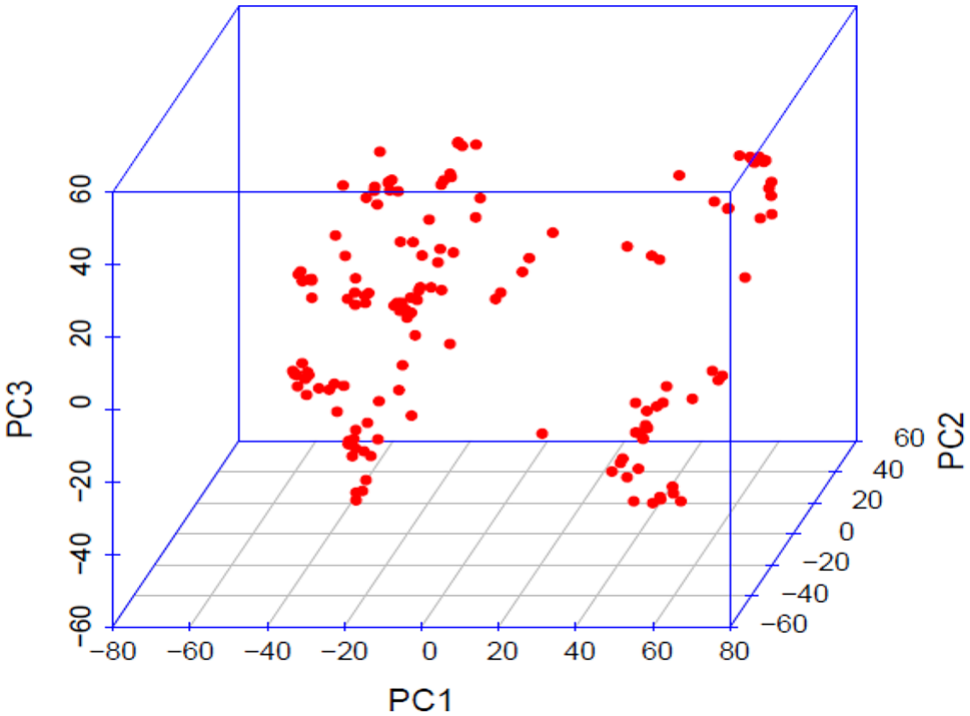
Principal component analysis for the 151 inbred lines

Principal component analysis results based on the 10,940 SNPs were in agreement with the population structure and NJ clustering, grouping the inbred lines into four subgroups (Fig. 5). The first and second PCs explained 79% and 13% of the SNP variation respectively.

## Discussion

Genetic diversity is imperative to provide a robust food security system capable of adapting to recurrent biotic and abiotic stresses. This is through identifying alleles that could be used as the source of novel genotypes which are high yielding and resilient for biotic and/or abiotic stresses as well as meeting the end-user demands in plant breeding. In the determination of the genetic diversity of a population, molecular markers are preferred since they are not influenced by environmental factors. In this study, 10,940 SNP markers were used to determine the genetic diversity and population structure of 151 maize inbred lines from different sources. In the present study, the PIC average was 0.33, a value found higher than that reported by Wu et al. (2016), Sserumaga et al. (2019), Ajala et al. (2019), and Adekemi et al. (2020). The PIC value in this study implies that the markers used are informative. According to Lemos et al. (2019), the polymorphism information content of a marker means the ability of this marker to detect the polymorphism among individuals of a population, and the higher that capacity, the greater its value. The average gene diversity, which is a degree of genetic diversity detected, in this study was higher than the GD in earlier studies done by Boakyewaa Adu et al. (2019) but similar to Sserumaga et al. (2019), Ajala et al. (2019), and Adekemi et al. (2020). According to Sserumaga et al. (2019), large GD estimates (> 0.5) between pairs of maize inbred lines imply the presence of a wide diversity to select from, thus leading to high levels of heterosis. This also means that most of the inbred lines used are unique and thus have the potential to contribute new alleles to the breeding program. The differences between the results from the current study and the findings of previous researchers can be explained by various factors such as differences in population size, different experimental materials, and the number of markers used in the different studies (Boakyewaa Adu et al. 2019).

The two complementary methods (admixture ancestry and hierarchical cluster analysis), used to determine the number of groups in this study, both clustered the inbred lines into four separate groups, thus implying there is a high degree of genetic diversity in the inbred lines evaluated in this study. Most of the inbred lines were clustered depending on their ancestry, selection history, and endosperm color (Figs. 3 and 4). However, some inbred lines were not clustered according to shared ancestry; this means that inbred lines obtained from the same source population do not necessarily have the same selection history (Boakyewaa Adu et al. 2019). This can be explained by the fact that the bi-parental populations used to develop the lines were from diverse sources, including temperate, tropical, and subtropical. This has been confirmed by various studies on tropical maize inbred lines which have reported diversity explained by the diverse makeup of the source germplasm used to develop these lines (Warburton et al. 2002, 2008; Semagn et al. 2012; Wu et al. 2016; Sserumaga et al. 2019). Furthermore, the variation observed can be due to other factors like selection, the breeding system used, and differences in the geographical origin of the source populations. It is important to note, in this study, the clustering of the lines into four groups was different between the neighbor-joining tree and the population structure; this can be attributed to the differences in the clustering algorithms and models employed by the two methods.

The low levels of heterozygosity ranging from 0.16 to 0.30 detected among the inbred lines within the four groups suggest that the SNP markers were efficient in creating homogeneous subpopulations. The F_ST_ values observed in the present study ranged from 0.24 to 0.65 and were high; these values were similar to those reported in previous studies on other tropical inbred lines by Boakyewaa Adu et al. (2019) and Sserumaga et al. (2019, 2020). These high F_ST_ values imply that the lines in the study are fixed and, therefore, grouped into genetically diverse groups; this makes them an important resource for genetic studies, and valuable for association mapping studies that require uniformity of inbred lines and genetic divergence. For the development of hybrids, it is advisable that crosses are made between parental lines from diverse clusters.

The AMOVA results (*P* < 0.001) also support the population differentiation. AMOVA revealed major molecular variance within populations rather than among populations implying a high genetic diversity among accessions (Sserumaga et al. 2021). According to Wang (2020), generally, outcrossing and long-lived plants have the most genetic variation within. Inbred lines from diverse subgroups possess unique alleles that could be beneficial in the breeding programs. The diversity observed among these maize inbred lines can be utilized and used to develop new lines. Given that some of these lines already have beneficial traits such as resistance to pests and diseases, and others are resistant to abiotic stresses such as drought, they could be good candidates for recycling. Such bi-parental crosses between genetically diverse inbred lines with good genetic potential will result in blends of several promising alleles at different loci leading to new lines with multiple stress resistance and higher yield potential.

## Conclusion

In this study, a considerable amount of genetic diversity was observed among the 151 inbred lines genotyped using the DArTseq genotyping platform. Four clusters were identified from this study. This genetic diversity can be utilized for future breeding progress to develop new maize varieties with desirable characteristics that are adapted to changing environments. This germplasm could also be used to produce a core collection, map population studies, and facilitate the identification of useful traits. The contrasting pair of inbred lines from different subpopulations could be used to create mapping populations to identify genes involved in disease, pest, and/or drought resistance in maize. Furthermore, the current genetic diversity information will be very useful for making more effective use of these inbred lines in tropical breeding programs for the development of open-pollinated varieties and/or hybrids, as well as for maintaining a broad genetic base that can be used to develop promising resistant and high yielding inbred lines.

## Supplementary Information

Below is the link to the electronic supplementary material.

Supplementary file1 (DOCX 26 KB)

## Data Availability

All the data and plant material are available from the corresponding author.
