# Supplementary material for: Genetic Variation Among Tropical Maize Inbred Lines from NARS and CGIAR Breeding Programs
Source: Plant Mol Biol Report. 2022 Sep 26;41(2):209–17. doi: 10.1007/s11105-022-01358-2 (PMC10160135; doi:10.1007/s11105-022-01358-2)
Supplement: Supplementary file 1 — Supplementary file1 (DOCX 26 KB) [file 11105_2022_1358_MOESM1_ESM.docx]

**Table 1: List of inbred lines used in the study including their pedigrees and origin**

| **No** | **Identification** | **Pedigree** | **Origin** |
| --- | --- | --- | --- |
| 1 | CKL05003 | [CML202/CML395-6]-B-B-2-1-B | CIMMYT |
| 2 | CKL05006 | [CML205/[EV7992#/EV8449-SR]C1F2-334-1(OSU8i)-1-1-Sn]-B-B-2-6-B-B-B*4 | CIMMYT |
| 3 | CKL05010 | [CML395-2/CML202]-B-3-3-3-B-B*4 | CIMMYT |
| 4 | CKL05017 | [CML387/CML390]-B-1-1-4-B-B*4 | CIMMYT |
| 5 | CKL05018 | [CML387/CML390]-B-1-2-1-B*4 | CIMMYT |
| 6 | CKL05019 | [CML390/CML197]-B-B-5-1-B*4 | CIMMYT |
| 7 | CKL05024 | [KILIMA(ST94)-S5:115/[M37W/ZM607#BF37SR...]]-B-B-3-5-B-B*4 | CIMMYT |
| 8 | CKL150020 | ((DTPWC9:@.F115.1.4.1.1.@.@.)*2/S1031Z)-B-50-1-4-1-BB | CIMMYT |
| 9 | CKL150027 | ((DTPWC9:@.F115.1.4.1.1.@.@.)*2/S1031Z)-B-74-1-3-1-BBB | CIMMYT |
| 10 | CKL150028 | ((DTPWC9:@.F115.1.4.1.1.@.@.)*2/S1031Z)-B-74-1-4-1-B*4 | CIMMYT |
| 11 | CKL150032 | ((DTPWC9:@.F115.1.4.1.1.@.@.)*2/S3512Z)-B-3-1-1-1-B*4 | CIMMYT |
| 12 | CKL150033 | ((DTPWC9:@.F115.1.4.1.1.@.@.)*2/S3512Z)-B-3-1-5-1-BBB | CIMMYT |
| 13 | CKL150038 | ((DTPWC9:@.F115.1.4.1.1.@.@.)*2/S3512Z)-B-6-1-2-1-B*4 | CIMMYT |
| 14 | CKL150039 | ((DTPWC9:@.F115.1.4.1.1.@.@.)*2/S3512Z)-B-14-1-3-1-B*4 | CIMMYT |
| 15 | CKL150041 | ((DTPWC9:@.F115.1.4.1.1.@.@.)*2/S3512Z)-B-19-1-3-1-B*4 | CIMMYT |
| 16 | CKL150042 | ((DTPWC9:@.F115.1.4.1.1.@.@.)*2/S3512Z)-B-20-1-1-1-B*4 | CIMMYT |
| 17 | CKL150043 | ((DTPWC9:@.F115.1.4.1.1.@.@.)*2/S3512Z)-B-25-1-1-1-B*4 | CIMMYT |
| 18 | CKL150050 | ((DTPWC9:@.F115.1.4.1.1.@.@.)*2/S3512Z)-B-50-1-2-1-BB | CIMMYT |
| 19 | CKL150053 | ((DTPWC9:@.F115.1.4.1.1.@.@.)*2/S3512Z)-B-65-1-1-1-B*4 | CIMMYT |
| 20 | CKL150054 | ((DTPWC9:@.F115.1.4.1.1.@.@.)*2/S3512Z)-B-65-1-2-1-B*4 | CIMMYT |
| 21 | CKL150061 | ((DTPWC9:@.F115.1.4.1.1.@.@.)*2/S3512Z)-B-88-1-2-1-BB | CIMMYT |
| 22 | CKL150062 | ((DTPWC9:@.F115.1.4.1.1.@.@.)*2/S3512Z)-B-88-1-4-1-BB | CIMMYT |
| 23 | CKL150063 | ((DTPWC9:@.F115.1.4.1.1.@.@.)*2/S3512Z)-B-89-1-1-1-BBB | CIMMYT |
| 24 | CKL150077 | ((DTPWC9:@.F16.1.1.1.1.@.@.)*2/S3512Z)-B-4-1-3-1-B*4 | CIMMYT |
| 25 | CKL150082 | ((DTPWC9:@.F16.1.1.1.1.@.@.)*2/S3512Z)-B-8-1-3-1-B*4 | CIMMYT |
| 26 | CKL150090 | ((DTPWC9:@.F16.1.1.1.1.@.@.)*2/S3512Z)-B-20-1-4-1-B*4 | CIMMYT |
| 27 | CKL150102 | ((P300C5S1B:@.2.3.2.1.1.1.2.@.@.)*2/S3512Z)-B-26-1-3-1-B*4 | CIMMYT |
| 28 | CKL150103 | ((P300C5S1B:@.2.3.2.1.1.1.2.@.@.)*2/S3512Z)-B-33-1-3-1-B*4 | CIMMYT |
| 29 | CKL150105 | (CML144*2/S3512Z)-B-29-1-2-1-B*4 | CIMMYT |
| 30 | CKL150109 | (CML144*2/S3512Z)-B-55-1-4-1-BBB | CIMMYT |
| 31 | CKL150110 | (CML144*2/S3512Z)-B-57-1-1-1-B*4 | CIMMYT |
| 32 | CKL150133 | (CML444*2/S3512Z)-B-32-1-4-1-B*4 | CIMMYT |
| 33 | CKL150135 | (CML444*2/S3512Z)-B-37-1-1-1-B*4 | CIMMYT |
| 34 | CKL150143 | (CML445*2/S3512Z)-B-4-1-3-1-BB | CIMMYT |
| 35 | CKL150162 | (CML78*2/S1920Z)-B-19-1-2-1-B*4 | CIMMYT |
| 36 | CKL150165 | (CML78*2/S1920Z)-B-66-1-2-1-B*4 | CIMMYT |
| 37 | CKL150525 | ((DTPWC9:@.F16.1.1.1.1.@.@.)*2/S3512Z)-B-11-1-3-1-BB | CIMMYT |
| 38 | CKL150551 | ((DTPWC9:@.F16.1.1.1.1.@.@.)*2/S3512Z)-B-18-1-1-1-BB | CIMMYT |
| 39 | CKL150557 | ((DTPWC9:@.F16.1.1.1.1.@.@.)*2/S3512Z)-B-20-1-1-1-BB | CIMMYT |
| 40 | CKL150559 | ((DTPWC9:@.F16.1.1.1.1.@.@.)*2/S3512Z)-B-20-1-3-2-BB | CIMMYT |
| 41 | CKL150594 | ((DTPWC9:@.F16.1.1.1.1.@.@.)*2/S3512Z)-B-8-1-1-1-BB | CIMMYT |
| 42 | CKL150791 | (CML395*2/S1031Z)-B-16-1-2-1-BB | CIMMYT |
| 43 | CKL150796 | (CML395*2/S3512Z)-B-18-1-1-2-BB | CIMMYT |
| 44 | CKL150797 | (CML395*2/S3512Z)-B-18-1-1-3-BB | CIMMYT |
| 45 | CKL150800 | (CML395*2/S3512Z)-B-18-1-3-2-BB | CIMMYT |
| 46 | CKL150807 | (CML395*2/S3512Z)-B-28-1-1-3-BB | CIMMYT |
| 47 | CKL150817 | (CML444*2/S3512Z)-B-14-1-5-1-BB | CIMMYT |
| 48 | CKL150836 | (CML444*2/S3512Z)-B-23-1-2-1-BB | CIMMYT |
| 49 | CKL150887 | (CML444*2/S3512Z)-B-6-1-2-1-BB | CIMMYT |
| 50 | CKL150888 | (CML444*2/S3512Z)-B-6-1-3-1-BB | CIMMYT |
| 51 | CKL150916 | (CML78*2/S1920Z)-B-19-1-2-2-BB | CIMMYT |
| 52 | CML444 | P43C9-1-1-1-1-1-BBBB | CIMMYT |
| 53 | DL12102 | ((KU1403 x 1368)-7-2-1-1-B-B/CML444)-B-3-2-3-2-2-1-7-B-B | CIMMYT |
| 54 | DL12130 | ((KU1403 x 1368)-7-2-1-1-B-B/CML444)-B-8-5-5-1-4-1-1-B-B | CIMMYT |
| 55 | DL12131 | ((KU1403 x 1368)-7-2-1-1-B-B/CML444)-B-8-5-5-1-4-2-1-B-B | CIMMYT |
| 56 | DL12132 | ((KU1403 x 1368)-7-2-1-1-B-B/CML444)-B-8-7-3-2-3-1-1-B-B | CIMMYT |
| 57 | DL12139 | ((KU1403 x 1368)-7-2-1-1-B-B/CML444)-B-8-8-2-1-4-2-4-B-B-B | CIMMYT |
| 58 | DL1298 | ((KU1403 x 1368)-7-2-1-1-B-B/CML444)-B-2-9-2-3-1-2-1-B-B | CIMMYT |
| 59 | DL141020 | (CKL05018/CML536)-B-37-1-1-3-2-B-B-B-B | CIMMYT |
| 60 | DL141025 | (CKL05018/CML536)-B-37-2-1-1-B-B-B-B-B | CIMMYT |
| 61 | DL141138 | (MAS[MSR/312]-117-2-2-1-B*6/ZM523A-16-2-1-1-BBB)-B-7-1-2-1-1-B | CIMMYT |
| 62 | DL141340 | (CKL05007/CIMCALI8843/S9243-BB-#-B-5-1-BB-4-1-3-2-B)-B-4-1-1-1-B-B-B-B-B | CIMMYT |
| 63 | DL141364 | (CKL05024/CIMCALI8843/S9243-BB-#-B-5-1-BB-2-3-1-B)-B-6-3-1-1-B-B-B-B-B | CIMMYT |
| 64 | DL141388 | ECAVL21-37-1-3-2-1-4-B-B-B-B | CIMMYT |
| 65 | DL141392 | ECAVL21-37-3-3-1-1-2-B-B-B-B | CIMMYT |
| 66 | DL14207 | (CKL05017/INTA/INTB-B-41-B-14-1-B)-B-25-3-1-8-2-B-B(CKL05017/INTA/INTB-B-41-B-14-1-B)-B-25-3-1-8-2-B-B | CIMMYT |
| 67 | DL14500 | ([CML444/CML395//DTPWC8F31-1-1-2-2-BB]-4-2-2-1-1-B*4/(9071xBabamgoyo)-3-1-BBB)-B-1-2-3-1-1-B-B | CIMMYT |
| 68 | DL14501 | ([CML444/CML395//DTPWC8F31-1-1-2-2-BB]-4-2-2-1-1-B*4/(9071xBabamgoyo)-3-1-BBB)-B-1-2-3-1-2-B-B-B-B-B | CIMMYT |
| 69 | DL14504 | ECAVL29-2-3-3-1-1-2-B-B | CIMMYT |
| 70 | DL14505 | ECAVL2/ECAVL17-#-2-3-3-3-1-B-B | CIMMYT |
| 71 | DL15140 | (CKL05003/La Posta Seq C7-F64-2-6-2-2-B-B -B)DH110-B-B-B--B | CIMMYT |
| 72 | DL1515 | (CKL05006/CML489)-B-13-1-2-B-B | CIMMYT |
| 73 | DL15193 | (CKL05006/LaPostaSeqC7-F71-1-2-1-2-B-B-B-B)DH11-B-B-B-B-B-B-B | CIMMYT |
| 74 | DL1558 | (CKL05003/CML444//CKL05003)DH5-B-B-B-B | CIMMYT |
| 75 | DL15643 | (CIMCALI8843/S9243-BB-#-B-5-1-BB-4-1-3-2-B/La Posta Seq C7-F78-2-1-1-1-B-B-B-B)-B-2-5-2-1-1-B-B | CIMMYT |
| 76 | DL15644 | (CIMCALI8843/S9243-BB-#-B-5-1-BB-4-1-3-2-B/La Posta Seq C7-F78-2-1-1-1-B-B-B-B)-B-2-5-2-2-1-B-B | CIMMYT |
| 77 | JPS25-1 | (NML85/(La Posta Seq C7-F96-1-2-1-1-B-B-B/CML444/CML444) DH-104-B-B-B)-B-1-1-1-B-B | NARO |
| 78 | JPS25-10 | (NML85/(La Posta Seq C7-F96-1-2-1-1-B-B-B/CML444/CML444) DH-104-B-B-B)-B-2-1-2-B-B | NARO |
| 79 | JPS25-11 | (NML85/(La Posta Seq C7-F96-1-2-1-1-B-B-B/CML444/CML444) DH-104-B-B-B)-B-2-1-4-B-B | NARO |
| 80 | JPS25-12 | (NML85/(La Posta Seq C7-F96-1-2-1-1-B-B-B/CML444/CML444) DH-104-B-B-B)-B-2-1-6-B-B | NARO |
| 81 | JPS25-13 | (NML85/(La Posta Seq C7-F96-1-2-1-1-B-B-B/CML444/CML444) DH-104-B-B-B)-B-3-1-2-B-B | NARO |
| 82 | JPS25-14 | (NML85/(La Posta Seq C7-F96-1-2-1-1-B-B-B/CML444/CML444) DH-104-B-B-B)-B-3-2-1-B-B | NARO |
| 83 | JPS25-15 | (NML85/(La Posta Seq C7-F96-1-2-1-1-B-B-B/CML444/CML444) DH-104-B-B-B)-B-4-1-2-B-B | NARO |
| 84 | JPS25-18 | (NML85/(La Posta Seq C7-F96-1-2-1-1-B-B-B/CML444/CML444) DH-104-B-B-B)-B-4-2-5-B-B | NARO |
| 85 | JPS25-2 | (NML85/(La Posta Seq C7-F96-1-2-1-1-B-B-B/CML444/CML444) DH-104-B-B-B)-B-1-1-2-B | NARO |
| 86 | JPS25-20 | (NML85/(La Posta Seq C7-F96-1-2-1-1-B-B-B/CML444/CML444) DH-104-B-B-B)-B-4-3-3-B-B | NARO |
| 87 | JPS25-22 | (NML85/(La Posta Seq C7-F71-1-2-1-2-B-B-B/CML312SR = MAS[MSR/312]-117-2-2-1-2-B*4-B-B-B-B/CML312SR) DH-10-B-B-B)-B-1-2-2-B-B | NARO |
| 88 | JPS25-23 | (NML85/(La Posta Seq C7-F71-1-2-1-2-B-B-B/CML312SR = MAS[MSR/312]-117-2-2-1-2-B*4-B-B-B-B/CML312SR) DH-10-B-B-B)-B-1-2-4-B-B | NARO |
| 89 | JPS25-24 | (NML85/(La Posta Seq C7-F71-1-2-1-2-B-B-B/CML312SR = MAS[MSR/312]-117-2-2-1-2-B*4-B-B-B-B/CML312SR) DH-10-B-B-B)-B-1-2-5-B-B | NARO |
| 90 | JPS25-25 | (NML85/(La Posta Seq C7-F71-1-2-1-2-B-B-B/CML312SR = MAS[MSR/312]-117-2-2-1-2-B*4-B-B-B-B/CML312SR) DH-10-B-B-B)-B-3-1-1-B-B | NARO |
| 91 | JPS25-26 | (NML85/(La Posta Seq C7-F71-1-2-1-2-B-B-B/CML312SR = MAS[MSR/312]-117-2-2-1-2-B*4-B-B-B-B/CML312SR) DH-10-B-B-B)-B-3-1-2-B-B | NARO |
| 92 | JPS25-27 | (NML85/(La Posta Seq C7-F71-1-2-1-2-B-B-B/CML312SR = MAS[MSR/312]-117-2-2-1-2-B*4-B-B-B-B/CML312SR) DH-10-B-B-B)-B-3-1-4-B-B | NARO |
| 93 | JPS25-28 | (NML85/(La Posta Seq C7-F71-1-2-1-2-B-B-B/CML312SR = MAS[MSR/312]-117-2-2-1-2-B*4-B-B-B-B/CML312SR) DH-10-B-B-B)-B-3-2-1-B-B | NARO |
| 94 | JPS25-29 | (NML85/(La Posta Seq C7-F71-1-2-1-2-B-B-B/CML312SR = MAS[MSR/312]-117-2-2-1-2-B*4-B-B-B-B/CML312SR) DH-10-B-B-B)-B-3-2-2-B-B | NARO |
| 95 | JPS25-3 | (NML85/(La Posta Seq C7-F96-1-2-1-1-B-B-B/CML444/CML444) DH-104-B-B-B)-B-1-1-3-B | NARO |
| 96 | JPS25-30 | (NML85/(La Posta Seq C7-F71-1-2-1-2-B-B-B/CML312SR = MAS[MSR/312]-117-2-2-1-2-B*4-B-B-B-B/CML312SR) DH-10-B-B-B)-B-3-2-4-B-B | NARO |
| 97 | JPS25-31 | (NML85/(La Posta Seq C7-F71-1-2-1-2-B-B-B/CML312SR = MAS[MSR/312]-117-2-2-1-2-B*4-B-B-B-B/CML312SR) DH-18-B-B-B)-B-1-1-4-B-B | NARO |
| 98 | JPS25-32 | (NML85/(La Posta Seq C7-F71-1-2-1-2-B-B-B/CML312SR = MAS[MSR/312]-117-2-2-1-2-B*4-B-B-B-B/CML312SR) DH-18-B-B-B)-B-1-1-5-B-B | NARO |
| 99 | JPS25-33 | (NML85/(La Posta Seq C7-F71-1-2-1-2-B-B-B/CML312SR = MAS[MSR/312]-117-2-2-1-2-B*4-B-B-B-B/CML312SR) DH-18-B-B-B)-B-1-2-1-B-B | NARO |
| 100 | JPS25-35 | (NML85/(La Posta Seq C7-F71-1-2-1-2-B-B-B/CML312SR = MAS[MSR/312]-117-2-2-1-2-B*4-B-B-B-B/CML312SR) DH-18-B-B-B)-B-2-1-4-B-B | NARO |
| 101 | JPS25-36 | (NML85/(La Posta Seq C7-F71-1-2-1-2-B-B-B/CML312SR = MAS[MSR/312]-117-2-2-1-2-B*4-B-B-B-B/CML312SR) DH-18-B-B-B)-B-2-2-2-B-B | NARO |
| 102 | JPS25-37 | (NML85/(La Posta Seq C7-F71-1-2-1-2-B-B-B/CML312SR = MAS[MSR/312]-117-2-2-1-2-B*4-B-B-B-B/CML312SR) DH-18-B-B-B)-B-3-1-1-B-B | NARO |
| 103 | JPS25-38 | (NML85/(La Posta Seq C7-F71-1-2-1-2-B-B-B/CML312SR = MAS[MSR/312]-117-2-2-1-2-B*4-B-B-B-B/CML312SR) DH-18-B-B-B)-B-3-1-6-B-B | NARO |
| 104 | JPS25-39 | (NML85/(La Posta Seq C7-F71-1-2-1-2-B-B-B/CML312SR = MAS[MSR/312]-117-2-2-1-2-B*4-B-B-B-B/CML312SR) DH-18-B-B-B)-B-7-1-3-B-B | NARO |
| 105 | JPS25-4 | (NML85/(La Posta Seq C7-F96-1-2-1-1-B-B-B/CML444/CML444) DH-104-B-B-B)-B-1-1-4-B | NARO |
| 106 | JPS25-40 | (NML85/(La Posta Seq C7-F71-1-2-1-2-B-B-B/CML312SR = MAS[MSR/312]-117-2-2-1-2-B*4-B-B-B-B/CML312SR) DH-18-B-B-B)-B-7-5-1-B-B | NARO |
| 107 | JPS25-41 | (NML85/(La Posta Seq C7-F71-1-2-1-2-B-B-B/CML312SR = MAS[MSR/312]-117-2-2-1-2-B*4-B-B-B-B/CML312SR) DH-18-B-B-B)-B-7-5-2-B-B | NARO |
| 108 | JPS25-42 | (NML85/(La Posta Seq C7-F71-1-2-1-2-B-B-B/CML312SR = MAS[MSR/312]-117-2-2-1-2-B*4-B-B-B-B/CML312SR) DH-18-B-B-B)-B-8-1-1-B-B | NARO |
| 109 | JPS25-43 | (NML85/(La Posta Seq C7-F71-1-2-1-2-B-B-B/CML312SR = MAS[MSR/312]-117-2-2-1-2-B*4-B-B-B-B/CML312SR) DH-18-B-B-B)-B-8-1-2-B-B | NARO |
| 110 | JPS25-44 | (NML85/(La Posta Seq C7-F71-1-2-1-2-B-B-B/CML312SR = MAS[MSR/312]-117-2-2-1-2-B*4-B-B-B-B/CML312SR) DH-18-B-B-B)-B-8-1-4-B-B | NARO |
| 111 | JPS25-46 | (NML85/(La Posta Seq C7-F71-1-2-1-2-B-B-B/CML312SR = MAS[MSR/312]-117-2-2-1-2-B*4-B-B-B-B/CML312SR) DH-18-B-B-B)-B-8-1-6-B-B | NARO |
| 112 | JPS25-48 | (NML85/(La Posta Seq C7-F71-1-2-1-2-B-B-B/CML312SR = MAS[MSR/312]-117-2-2-1-2-B*4-B-B-B-B/CML312SR) DH-18-B-B-B)-B-8-1-8-B-B | NARO |
| 113 | JPS25-49 | (NML85/(La Posta Seq C7-F71-1-2-1-2-B-B-B/CML312SR = MAS[MSR/312]-117-2-2-1-2-B*4-B-B-B-B/CML312SR) DH-18-B-B-B)-B-8-2-1-B-B | NARO |
| 114 | JPS25-5 | (NML85/(La Posta Seq C7-F96-1-2-1-1-B-B-B/CML444/CML444) DH-104-B-B-B)-B-1-1-5-B | NARO |
| 115 | JPS25-51 | (NML85/(La Posta Seq C7-F71-1-2-1-2-B-B-B/CML395/CML395) DH-21-B-B-B)-B-1-1-1-B-B | NARO |
| 116 | JPS25-52 | (NML85/(La Posta Seq C7-F71-1-2-1-2-B-B-B/CML395/CML395) DH-21-B-B-B)-B-1-1-3-B-B | NARO |
| 117 | JPS25-53 | (NML85/(La Posta Seq C7-F71-1-2-1-2-B-B-B/CML395/CML395) DH-21-B-B-B)-B-4-1-2-B-B | NARO |
| 118 | JPS25-54 | (NML85/(La Posta Seq C7-F71-1-2-1-2-B-B-B/CML444/CML444) DH-49-B-B-B)-B-1-1-2-B-B | NARO |
| 119 | JPS25-55 | (NML97/(La Posta Seq C7-F96-1-2-1-1-B-B-B/CML444/CML444) DH-104-B-B-B)-B-3-1-3-B-B | NARO |
| 120 | JPS25-56 | (NML97/(La Posta Seq C7-F71-1-2-1-2-B-B-B/CML395/CML395) DH-65-B-B-B)-B-1-4-1-B-B | NARO |
| 121 | JPS25-57 | (NML97/(La Posta Seq C7-F71-1-2-1-2-B-B-B/CML395/CML395) DH-65-B-B-B)-B-2-5-3-B-B | NARO |
| 122 | JPS25-58 | (NML97/(La Posta Seq C7-F71-1-2-1-2-B-B-B/CML444/CML444) DH-49-B-B-B)-B-1-2-2-B-B | NARO |
| 123 | JPS25-6 | (NML85/(La Posta Seq C7-F96-1-2-1-1-B-B-B/CML444/CML444) DH-104-B-B-B)-B-1-1-7-B | NARO |
| 124 | JPS25-60 | (NML88/(La Posta Seq C7-F96-1-2-1-1-B-B-B/CML444/CML444) DH-104-B-B-B)-B-2-1-1-B-B | NARO |
| 125 | JPS25-61 | (NML88/(La Posta Seq C7-F71-1-2-1-2-B-B-B/CML312SR = MAS[MSR/312]-117-2-2-1-2-B*4-B-B-B-B/CML312SR) DH-5-B-B-B)-B-3-3-1-B-B | NARO |
| 126 | JPS25-62 | (NML88/(La Posta Seq C7-F71-1-2-1-2-B-B-B/CML312SR = MAS[MSR/312]-117-2-2-1-2-B*4-B-B-B-B/CML312SR) DH-10-B-B-B)-B-1-1-1-B-B | NARO |
| 127 | JPS25-63 | (NML88/(La Posta Seq C7-F71-1-2-1-2-B-B-B/CML312SR = MAS[MSR/312]-117-2-2-1-2-B*4-B-B-B-B/CML312SR) DH-10-B-B-B)-B-3-1-3-B-B | NARO |
| 128 | JPS25-64 | (NML88/(La Posta Seq C7-F71-1-2-1-2-B-B-B/CML312SR = MAS[MSR/312]-117-2-2-1-2-B*4-B-B-B-B/CML312SR) DH-10-B-B-B)-B-3-2-1-B-B | NARO |
| 129 | JPS25-65 | (NML88/(La Posta Seq C7-F71-1-2-1-2-B-B-B/CML444/CML444) DH-49-B-B-B)-B-1-1-2-B-B | NARO |
| 130 | JPS25-7 | (NML85/(La Posta Seq C7-F96-1-2-1-1-B-B-B/CML444/CML444) DH-104-B-B-B)-B-1-1-8-B | NARO |
| 131 | JPS25-8 | (NML85/(La Posta Seq C7-F96-1-2-1-1-B-B-B/CML444/CML444) DH-104-B-B-B)-B-1-1-10-B | NARO |
| 132 | JPS25-9 | (NML85/(La Posta Seq C7-F96-1-2-1-1-B-B-B/CML444/CML444) DH-104-B-B-B)-B-2-1-1-B | NARO |
| 133 | JPS26-1 | POBLAC21CO/NML 56-1-1-1 | NARO |
| 134 | JPS26-112 | POBLAC2ICO/WL 118-17-1-1-1 | NARO |
| 135 | JPS26-119 | POBLAC2ICO/WL 118-17-3-2-1 | NARO |
| 136 | JPS26-125 | POBLAC2ICO/WL 118-17-4-3-1 | NARO |
| 137 | JPS26-26 | POBLAC21CO/NML 56-3-3-5 | NARO |
| 138 | JPS26-37 | POBLAC21CO/NML 56-4-3-1 | NARO |
| 139 | JPS26-4 | POBLAC21CO/NML 56-1-2-3 | NARO |
| 140 | JPS26-43 | POBLAC21CO/NML 56-6-2-2 | NARO |
| 141 | JPS26-5 | POBLAC21CO/NML 56-1-3-1 | NARO |
| 142 | JPS26-60 | POBLAC21CO/NML 56-8-2-2 | NARO |
| 143 | JPS26-68 | POBLAC21CO/NML 56-12-2-3 | NARO |
| 144 | JPS26-77 | POBLAC21CO/NML 85-9-3-1 | NARO |
| 145 | JPS26-81 | POBLAC2ICO/NML 89-2-2-1 | NARO |
| 146 | JPS26-82 | POBLAC2ICO/NML 89-2-2-2 | NARO |
| 147 | JPS26-86 | POBLAC2ICO/NML 89-5-2-2 | NARO |
| 148 | WL118-10 | [WEEVIL/CML202]-B-7-B-B-B-B | NARO |
| 149 | WL118-3 | [WEEVIL/CML387]-B-8-B-B-B-B | NARO |
| 150 | WL429-24 | [WEEVIL/CML312]-B-1-B-B-B-B | NARO |
| 151 | WL429-38 | [WEEVIL/CML389]-B-17-B-B-B-B | NARO |
